# Direct Measurement of the Local Density of Optical States in the Time Domain

**DOI:** 10.1021/acsphotonics.3c00781

**Published:** 2023-08-01

**Authors:** Stan E. T. ter Huurne, Djero B. L. Peeters, Jose A. Sánchez-Gil, Jaime Gómez Rivas

**Affiliations:** † Department of Applied Physics and Science Education, and Eindhoven Hendrik Casimir Institute, Eindhoven University of Technology, P.O. Box 513, Eindhoven 5600 MB, The Netherlands; ‡ Instituto de Estructura de La Materia, Consejo Superior de Investigaciones Científicas (IEM-CSIC), Serrano 121, Madrid 28006, Spain

**Keywords:** local density of optical states, terahertz spectroscopy, near-field, microscopy, time-domain spectroscopy

## Abstract

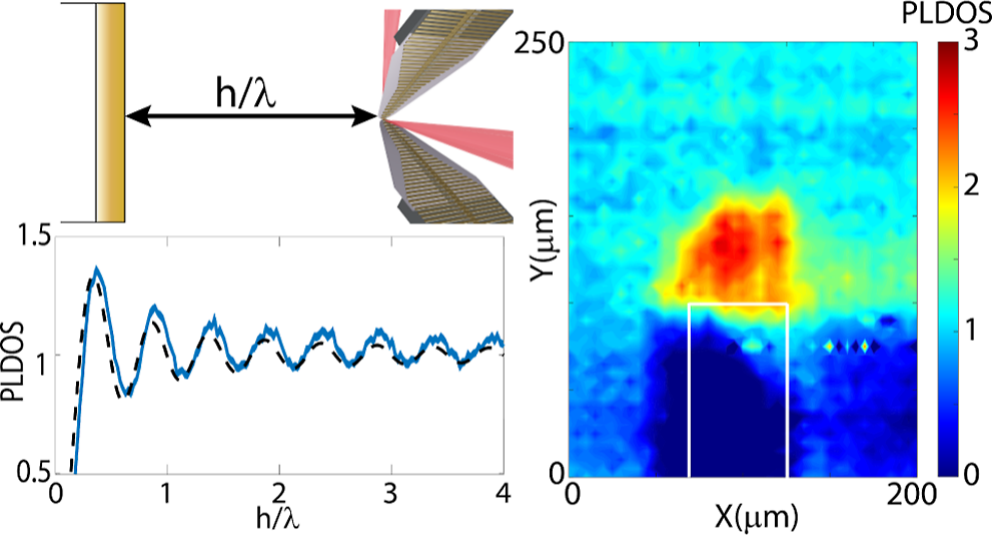

One of the most fundamental and relevant properties of a photonic system is the local density of optical states (LDOS) as it defines the rate at which an excited emitter dissipates energy by coupling to its surrounding. However, the direct determination of the LDOS is challenging as it requires measurements of the complex electric field of a point dipole at its own position. We introduce here a near-field setup which can measure the terahertz electric field amplitude at the position of a point source in the time domain. From the measured amplitude, the frequency-dependent imaginary component of the electric field can be determined and the LDOS can be retrieved. As a proof of concept, this setup has been used to measure the partial LDOS (the LDOS for a defined dipole orientation) as a function of the distance to planar interfaces made of gold, InSb, and quartz. Furthermore, the spatially dependent partial LDOS of a resonant gold rod has been measured as well. These results have been compared with analytical results and simulations. The excellent agreement between measurements and theory demonstrates the applicability of this setup for the quantitative determination of the LDOS in complex photonic systems.

## Introduction

Changes in the photonic environment of a point dipole govern the local density of optical states (LDOS), which is a measure of the number of modes per unit volume to which the excited dipole can decay. The number of available optical modes defines how efficiently the dipole can emit radiation.^1−6^ The presence of resonant structures changes the photonic environment and the LDOS, altering how light sources decay.^7−10^ The spatially dependent LDOS can be engineered by altering the surrounding of the dipole with resonant structures, increasing or decreasing the number of available modes. The LDOS is, therefore, an important quantity for fundamental processes, such as energy transfer, light–matter interaction, or single-photon emission, and for applications, such as solid-state lighting, solar cells, lasers, and optical sensors.^11−22^ The LDOS is not only important in the field of photonics but also has implications for any wave phenomena, such as sound waves or in nuclear physics.^23,24^

Changes in the decay rate of a point dipole due to a change in the photonic surrounding were initially demonstrated by Drexhage, where the fluorescence lifetime of a europium–dibenzoylmethane complex was measured as a function of the distance to a mirror.^25^ Ever since this discovery, there has been a significant effort to modify the properties of emitters by altering their coupling to the surrounding.^7,8,26−28^ Arguably, the manipulation of the LDOS has driven the field of nanophotonics during the past decades, with notable examples of photonics crystals and optical nanoantennas.^29−34^ Generally, the LDOS is determined by investigating the decay rate and lifetime of optical sources, which is altered by the LDOS, or through electron energy loss spectroscopy.^35−38^ However, direct measurements of the LDOS remain challenging as these measurements require the determination of the imaginary component of the electric field emitted by the dipole at its own position.

In this article, we use a novel local excitation and detection double near-field setup capable of measuring linearly polarized broadband terahertz (THz) pulses in the time domain.^39,40^ Measuring the electric field amplitude in the time domain allows for the determination of the complex electric field and thus its imaginary component. The LDOS for a single dipole orientation, called the partial LDOS (PLDOS), can be directly obtained from such measurements if the distance between the THz emitter probe and the detector is considerably smaller than the wavelength of the emitted THz radiation. The only limitations of the technique are those imposed by the size of the THz emitter probe, which needs to be much smaller than the wavelength to approximate it as a point dipole, and the distance between the emitter and detector probes. The technique can be applied to any surface (dielectric, semiconductor, or metal). Proof-of-concept measurements are performed by measuring the electric field in the time domain as a function of distance from several planar interfaces, reproducing the Drexhage experiment. Using this method, we also map the PLDOS close to a gold-rod resonator. These measurements are supported by calculations and simulations, showing an excellent agreement. This method allows the first direct measurement of the spatially dependent PLDOS in complex photonic media and will help to design and understand resonant systems that can control and improve light–matter interaction.

## Experimental Setup and PLDOS

The setup that has been developed for the direct measurement of the PLDOS uses a unique local excitation and detection THz time-domain spectroscopy (THz-TDS) technique. With THz-TDS, a femtosecond optical laser pulse is used to generate a broadband single-cycle THz pulse in the THz frequency range. Microstructured photoconductive antennas (MPCAs) are used in our setup for the excitation and detection of the THz pulse. These MPCAs are made up of a 1 μm thick piece of defect-rich low-temperature-grown GaAs on which gold electrodes are placed. The gap between these electrodes defines the photoconductive gap of the antenna, which is approximately 10 μm for the MPCAs. Carriers are generated in this gap by the femtosecond laser pulses with a power of approximately 3 mW. By applying a DC bias voltage of 10 V between these electrodes, the carriers are accelerated, resulting in the emission of short electromagnetic pulses in the THz frequency range. These pulses are polarized along the direction of the photoconductive gap. For detection, the free carriers generated in the MPCA detector by femtosecond laser pulses are not accelerated by an applied bias voltage. Instead, the electric field amplitude of the THz pulses acts as a time-dependent bias that accelerates the free carriers, resulting in a current proportional to the amplitude and polarization of the THz electric field. By changing the relative arrival time between the femtosecond laser pulses used for excitation and detection with the use of a beam splitter and a delay stage for one of the optical paths, the THz amplitude can be measured in the time-domain and the pulse retrieved (a schematic of the setup can be found in the Supporting Information).

**Figure 1:**
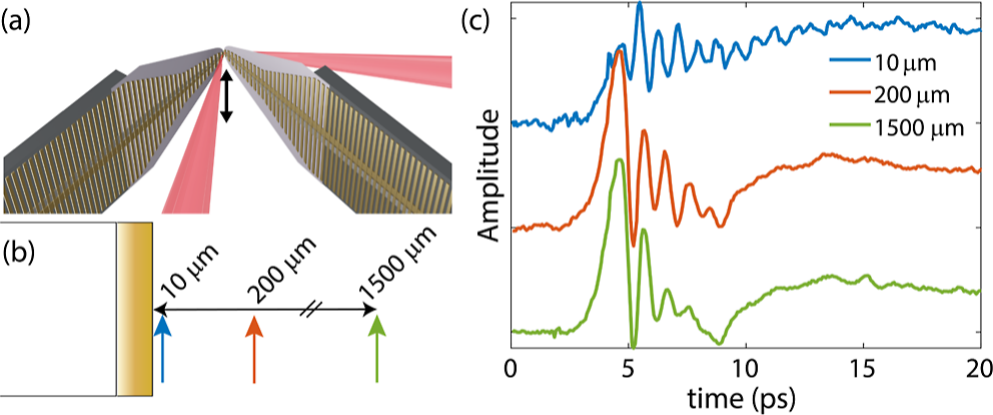
(a) Schematic representation of the local source and detector, consisting of two MPCAs positioned 10 μm apart. The red areas illustrate the fs beams, focused to a size of 10 μm, used for the generation and detection of the THz pulses. The black arrow indicates the orientation of the polarization. (b) Schematic representation of the performed experiment. The distance between the interface and the source is varied; the source is polarized parallel with the surface. (c) Transient signals measured at three distances from a gold mirror given in the legend.

To enable the determination of the PLDOS, both the source and detector are miniaturized. Due to the size of the MPCA, much smaller than the wavelength of THz radiation (λ/30 at 1 THz), the source can be approximated as a radiating dipole.^39^ The detector is positioned as close as possible to the emitter at a distance significantly smaller than the wavelength, typically 5 μm, to measure the emitted electric field at the position of the source. The local source and detector with the optical laser pulses and the polarization of the THz pulse are schematically shown in Figure 1a.

The LDOS at position ***r***_0_ and frequency ω [ρ(***r***_0_, ω)] can be calculated from the electromagnetic dyadic Green function ***G⃡***,^4,28^ defining the electric field at position ***r*** produced by a point source at ***r***_0_
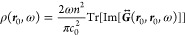
1where *n* is the refractive index of the medium and *c*_0_ is the speed of light in vacuum. The Green function is a 3 × 3 matrix with components *i*,*j*, where *i*,*j* = *x*,*y*,*z*. In eq 1, the LDOS is given by the trace of the imaginary component of the Green function at ***r***_0_. It is thus given by the imaginary component of the electric field produced by the source at its own position. The columns of the dyadic Green function indicate the three orthogonal orientations of the dipole moment, *x*, *y*, and *z*, respectively. Assuming that the electric field is produced by a point dipole, the Green function is related to the electric field ***E***(***r***) of the electric dipole ***p*** at ***r***_0_ by^28^
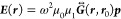
2where μ_0_ is the vacuum permeability and μ_1_ is the permeability of the medium. Therefore, the LDOS can be determined directly by measuring the imaginary component of the electric field amplitude at ***r***_0_.

The rate of energy dissipation of the dipole can be determined from both the intrinsic and scattered electric field amplitudes
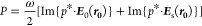
3

By dividing eq 3 by the intrinsic energy dissipation rate *P*_0_, we retrieve the relative importance of the scattered field. The change in the energy dissipation rate, which is equal to the change in the LDOS, is determined by the ratio of the imaginary components of the scattered and intrinsic electric field amplitudes along their dipole orientation
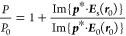
4

Therefore, we can directly measure the change along the orientation of the dipole source by referencing the imaginary component of the scattered field on the electric field amplitude without the presence of the scattered radiation, retrieving the partial LDOS (PLDOS).

## Results and Discussion

### PLDOS of a Planar Interface

The performed experiments are similar to the seminal work by Drexhage,^25^ but instead of measuring the decay rate of a point dipole, we measure the electric field from a broadband microstructured THz source in the time-domain at the position of the source. In this experiment, the field is measured as a function of the distance from a planar interface, whose reflection influences the PLDOS. This influence can increase or decrease the PLDOS, depending on the distance to the interface and the wavelength of the radiation.^5,6^ These experiments have been performed with different planar interfaces consisting of a 100 nm thick gold mirror, an InSb wafer, and a quartz substrate. The fabrication method of the gold mirror is given in the Supporting Information.

The colored arrows in Figure 1b indicate several distances from the interface and the polarization direction. The source is polarized parallel to the interface. The measured transients at three distances are shown in Figure 1c for the case of the gold mirror. At a distance of 10 μm (blue transient), the source is very close to the mirror (relative to the wavelength), and the electric field amplitude is considerably quenched. Due to the reduction of the PLDOS close to the mirror, the THz pulse is stretched in time, increasing the time it takes for the source to decay. At a distance of 200 μm (red transient), the electric field amplitude is much larger and the pulse is shorter in time. The reflection from the mirror arrives back at the source 1.33 ps after emission, interfering with the radiation still being emitted by the source. This interference results in several oscillations which are absent at a larger distance (green transient). At the farthest distance of 1500 μm, the source decays before the reflection on the mirror reaches the emitter again, which takes 10 ps. This reflection can be seen as a small oscillation at 15 ps in the green transient of Figure 1c.

**Figure 2:**
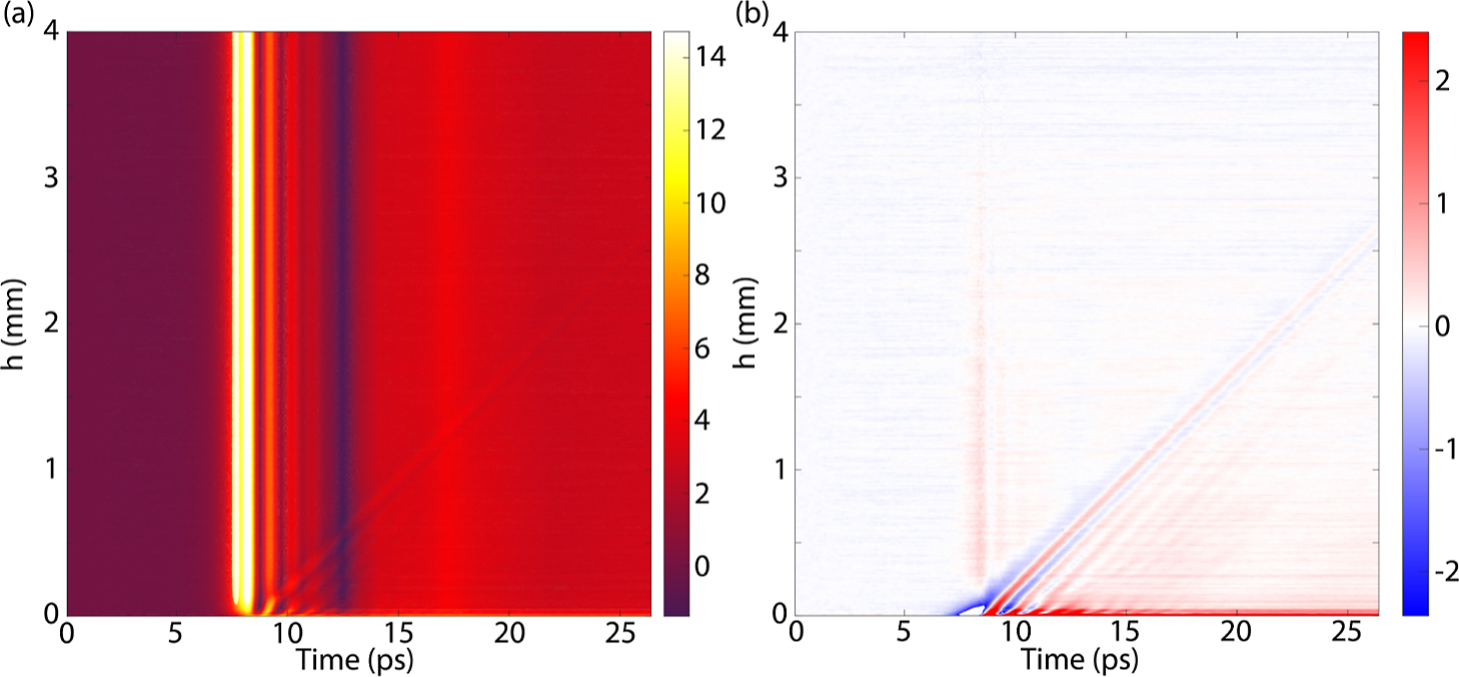
(a) Map of the electric field amplitude as a function of time and distance to the gold mirror, *h*. The diagonal signal is the reflection, which is delayed by increasing the distance to the mirror. (b) Map of the electric field amplitude difference as a function of time and distance to the mirror. The electric field amplitude measured at a distance of 4 mm from the mirror is subtracted at each position every time to clearly illustrate the reflection from the mirror. The plot is saturated at distances shorter than 70 μm to ensure a clear visualization of the reflection.

The three measurements of Figure 1 belong to a scan taken at distances varying from 0 to 4000 μm. This scan is shown as a function of time and distance to the interface in Figure 2a. At large distances away from the mirror, the electric field amplitude oscillates a few times as a function of time. Starting at 2.7 mm away from the mirror, the reflection becomes apparent as a diagonal, arriving earlier when moving closer to the mirror. At approximately 500 μm, the reflection starts to interfere with the emitted radiation, becoming a single convoluted pulse. The PLDOS is determined by the phase relation and the relative amplitude between the emitted signal and the reflection. To signify the influence of the reflected radiation from the mirror, we can focus on that signal in the time domain. This reflection can be made more apparent by subtracting the transient signal at a long distance from the interface, where the reflection arrives at times later than the measured time window (longer than 2.7 mm). The resulting map reveals the influence of the mirror on the emitted radiation as a function of time and distance, as shown in Figure 2b. In this figure, the reflection from the mirror is clearly visible, as well as the increase in the amplitude of this reflection when moving the source closer to the mirror. For distances shorter than 70 μm, the plot in Figure 2b is saturated. Otherwise, the quenching of the electric field amplitude at longer distances would not be visible.

**Figure 3:**
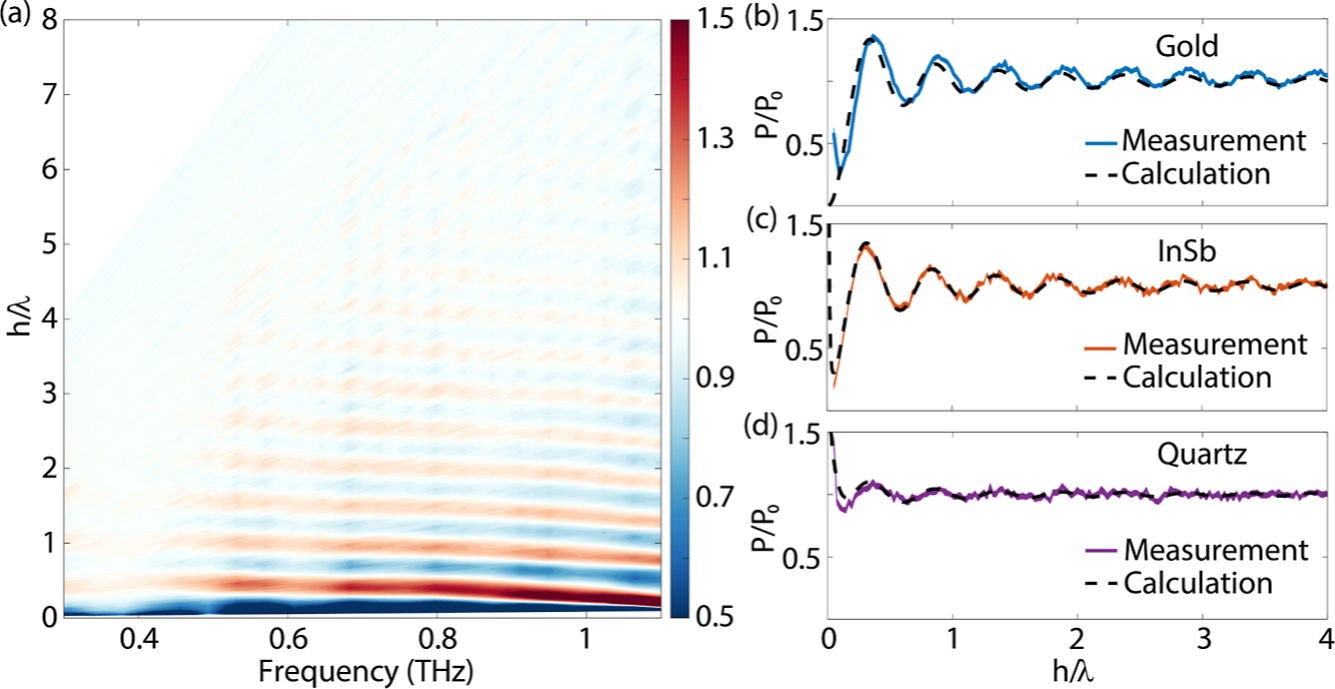
(a) Imaginary component of the normalized Fourier transform of the electric field amplitude as a function of frequency and distance to the mirror relative to the wavelength. Comparison between the measured PLDOS enhancement and analytical result at 0.76 THz as a function of distance from an interface of (b) gold, (c) InSb, and (d) quartz. The line widths of the measurement curves correspond to the standard deviation of the mean.

By Fourier transforming the transient signals, the electric field amplitude can be determined for every frequency at each distance. Since we measure in the time domain, we obtain the complex electric field amplitude and investigate the imaginary component. To ensure that the relative phase in each measurement is the same, the Fourier-transformed complex amplitude is multiplied by an offset. This offset is given by , where ω is the angular frequency and *t*_peak_ is the time from the start of the measurement to the peak THz amplitude (approximately 8 ps, as shown in Figure 2a). This offset ensures that the phase becomes relative with respect to the time of the THz peak amplitude, instead of the chosen starting time of the measurement. By normalizing this amplitude to measurements where the reflection on the mirror is not relevant (at distances longer than 2.7 mm away from the interface), we can investigate how the radiative power is enhanced or suppressed by the modified PLDOS at different distances from the interface (see eq 3). A map of the imaginary component of the electric field amplitude as a function of frequency and distance to the interface relative to the wavelength is shown in Figure 3a, starting from a distance of 25 μm. In this figure, clear oscillations of the PLDOS are visible as a function of the distance between the MPCAs and the interface. The oscillatory behavior of the PLDOS is characteristic of the self-action of the emitted radiation by the point dipole on itself due to the reflection at the interface.

The measurements are compared with analytical results,^28^ as shown in Figure 3b for 0.76 THz. There is an excellent agreement between the measurements and calculations until the emitter gets too close to the gold interface. Close to this interface, the measured electric field amplitude gets quenched due to the reduced PLDOS. Fourier transforming this small signal results in a small complex electric field amplitude with noise in the phase. This noise disrupts the distribution of the complex electric field amplitude in the real and imaginary components and yields an inaccurate result on the PLDOS.

Similar experiments have been performed at interfaces made of InSb and quartz. After performing the same analysis as for the gold interface, the PLDOS enhancement as a function of distance is determined and shown in Figure 3c,d at a frequency of 0.76 THz, together with the analytical results. The measurements start at a distance of 20 μm from the interface. The agreement between measurements and theory is excellent, both for InSb and quartz. Due to this agreement, we are confident that our measurement technique accurately determines the change of the PLDOS due to the photonic surroundings of the source and that the MPCAs do not alter this PLDOS significantly. In the next section, we investigate how the PLDOS is modified by a resonant structure.

### PLDOS of a Gold Rod Resonator

The structure that will be investigated in this section is a gold rod on a quartz substrate with a length and width of 200 and 40 μm, respectively, and a height of 100 nm. The fabrication method of the gold rod is given in the Supporting Information. This structure acts as a resonator and has its fundamental half-wavelength (λ/2) resonance along the long axis of the rod in the THz frequency range (see the Supporting Information for a total-field/scattered-field simulation of the resonance). By mapping the emission from the MPCA at its own position and for different locations close to the rod, the coupling between the source and the resonant structure can be determined in terms of the PLDOS.

**Figure 4:**
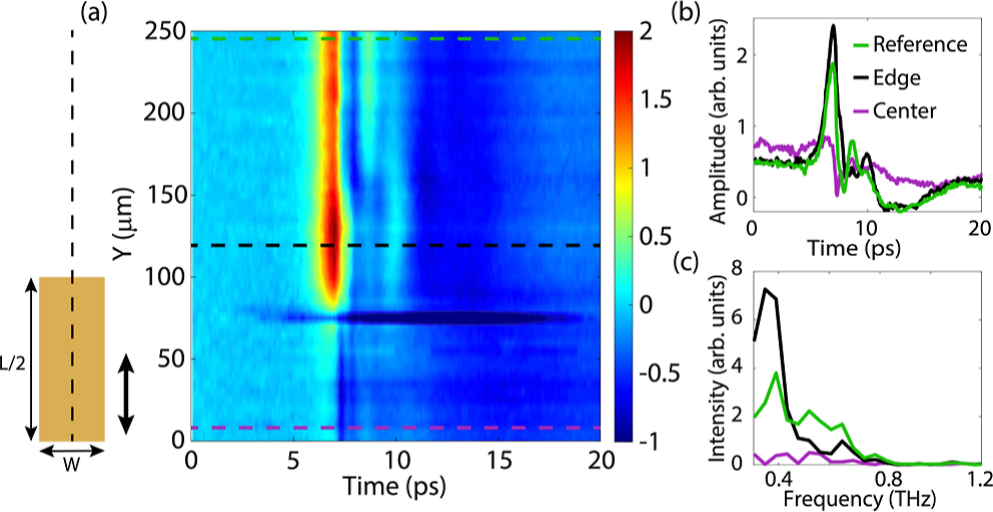
(a) Electric field amplitude as a function of time along the top half of the long axis of the gold rod. The dashed horizontal lines indicate the three distinct regions. (b) Electric field amplitude close to the gold rod resonator as a function of time at three different positions. (c) Intensity of the Fourier transform of the three transients, showing an enhancement for the edge of the rod at 0.4 THz and quenching at the center of the rod.

The first measurement consists of a line scan of transients close to the surface (a height of approximately 50 μm) and along the long axis of the gold rod. The source and detector are polarized along this axis, such that they can couple to the λ/2 resonance. We will only consider the data from the top half of the rod. Due to symmetry, we expect the same response for both halves. However, due to a slight misalignment between the source and detector, there is also an asymmetry in the measurements (the data of the bottom half is shown in the Supporting Information). A map of this measurement is shown in Figure 4a, where three distinct regions are visible. The first region is far away from the rod, where the transient signal is detected without the influence of the rod. In Figure 4a, this region is at the edge of the measurement (at *Y* = 200–250 μm). The second region is at the edges of the rod, where an enhanced electric field amplitude is visible, as well as additional time oscillations due to coupling with the rod and the modified PLDOS. The third region is on the rod, where the electric field amplitude is quenched, which severely reduces the emission from the source. This region behaves similarly to the case where the source is very close to a gold interface, as discussed in the previous section.

We focus now on the three regions by describing the transient signals in more detail. The transients of three measurements are shown in Figure 4b, with the positions of the measurements indicated by the colored dashed lines in Figure 4a. The first transient is the reference measurement far from the rod, the second transient is at the edge of the gold rod, and the third transient is at the center of the gold rod. The transient amplitude is enhanced considerably at the edge of the rod, indicating that the source modifies its emission due to the change in the PLDOS. A significant quenching of the emitted radiation is visible on the center of the rod. The spectra of the emitted radiation are very interesting since these spectra should map the resonant response of the rod. The intensities of the Fourier transforms (power spectra) of the three transients are shown in Figure 4c. At the edge of the gold rod, the emitted radiation is enhanced considerably at frequencies from 0.2 to 0.5 THz. These frequencies correspond to the λ/2 resonance, which is determined to be at 0.4 THz, as shown in the Supporting Information. On the rod, the emitted radiation gets quenched for all frequencies. These spectra illustrate that the in-plane *Y*-field component is resonantly enhanced at the edge of the rod due to the coupling to the λ/2 resonance.

**Figure 5:**
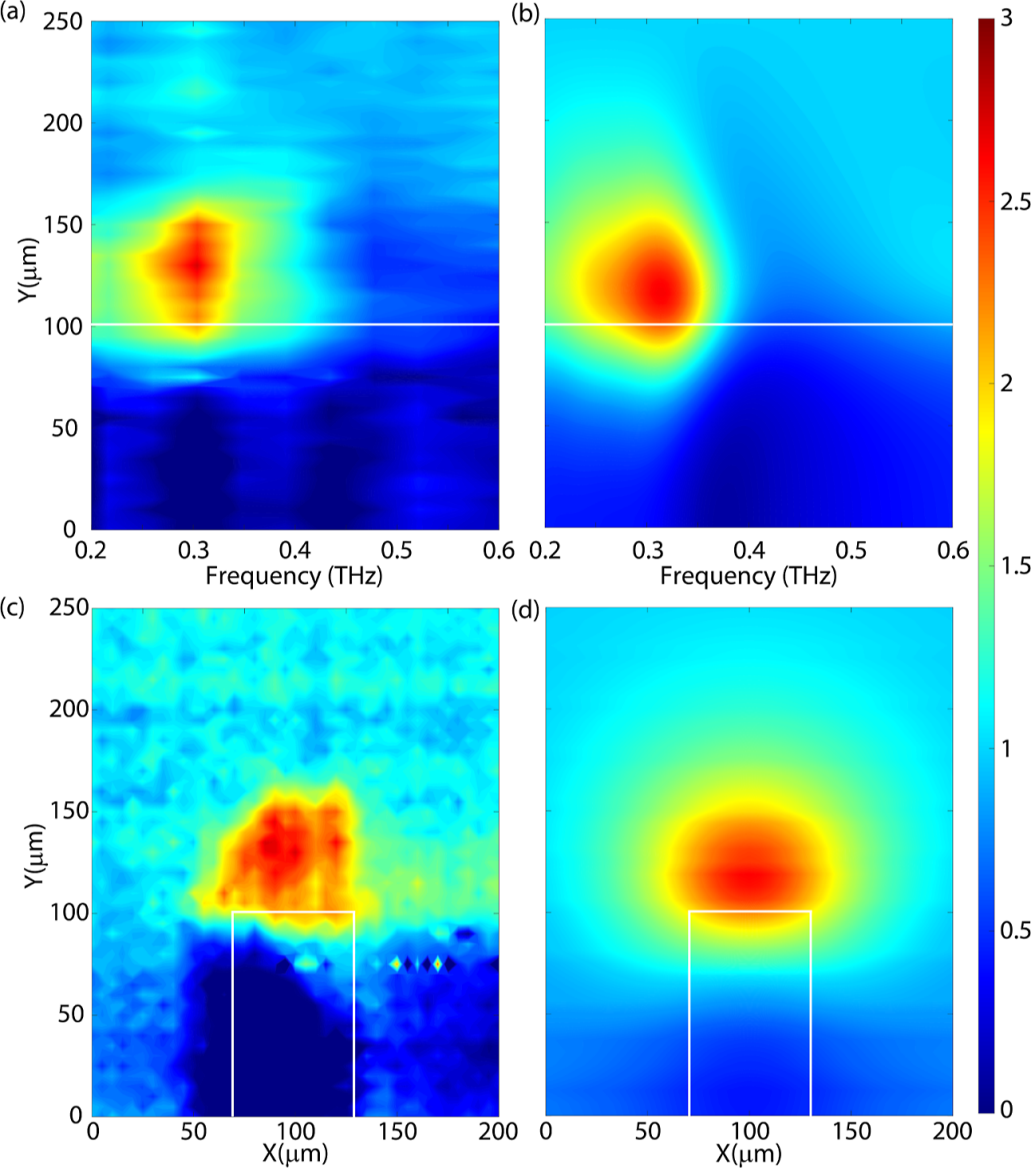
Map of the (a) measured and (b) simulated PLDOS of a gold rod on a quartz substrate normalized by the PLDOS of the quartz substrate as a function of frequency along the direction of the long axis of the rod. Spatial map of the (c) measured and (d) simulated PLDOS at 0.31 THz normalized by the PLDOS measured on the quartz substrate. The white lines indicate the edges of the rod.

We are interested in the PLDOS. Therefore, we investigate the normalized imaginary component of the electric field amplitude with and without the influence of the gold resonator. A map of the frequency-dependent normalized PLDOS along the long axis of the rod is shown in Figure 5a. These measurements are normalized to the average value far away from the rod (at *Y* = 250 μm) to differentiate the influence of the rod from the quartz substrate. On the rod, the PLDOS is close to 0 and is even negative for some frequencies/positions. Negative values of the PLDOS are attributed to a very low signal, resulting in a large noise. In Figure 5a, the negative values have been set to 0. At the edge of the rod, a PLDOS enhancement is detected close to the resonance frequency, indicating that the MPCA can couple to the resonator from its edges and radiate more efficiently. Away from the resonance frequency, for example, at 0.5–0.6 THz, the PLDOS at the edge becomes smaller than 1. Far away from the rod, the PLDOS enhancement converges to the expected value of 1.

These experimental results are compared to finite-difference time-domain (FDTD) simulations using Lumerical. In these simulations, a point dipole source is positioned 50 μm above the quartz substrate with the gold rod. This point dipole emits a polarized broadband pulse in the THz frequency range, which interacts with the surroundings. The radiated power is recorded to determine the PLDOS. By mapping the emission of the point dipole across the surface, the position- and frequency-dependent emission from the source can be simulated. A similar line scan as the experiment is shown in Figure 5b. Here, the simulation is normalized to the value at *Y* = 250 μm to reference the influence of the quartz substrate. The simulation shows a similar quantitative and qualitative enhancement of the PLDOS at the edges of the rod and a quenching of the PLDOS on the rod.

We have measured a spatial map surrounding the top half of the gold rod resonator to visualize the enhancement and reduction of the PLDOS more clearly. The spatial map of the PLDOS at the frequency of maximum enhancement (at 0.31 THz) is shown in Figure 5c. Similarly to the line scan measurement, the PLDOS is normalized to the average value far away from the rod (at *Y* = 250 μm). The PLDOS is quenched on the gold rod and at the sides of the long axis of the gold rod. At the edge, the PLDOS is enhanced by approximately a factor of 2.5 and is shaped as a semi-circle. The enhancement reaches approximately 50 μm away from the edge of the rod. About 100 μm away from the edges, the PLDOS is constant at a value of 1.

The measurements are compared with FDTD simulations under similar conditions, i.e., at the same frequency and at a height of 50 μm from the gold rod. A map of the simulated PLDOS is shown in Figure 5d. The simulations match well with the experiments, with the PLDOS quenched on the rod and at the sides of the long axis and an enhancement on the edge.

## Conclusions and Outlook

In conclusion, we have measured the PLDOS of photonic systems by retrieving the complex electric field at the position of a dipole source. Results of the PLDOS are shown as a function of the distance to three different planar interfaces (gold, InSb, and quartz), as well as for a gold rod resonator. The measurements have been compared with the analytical calculations and FDTD simulations, showing an excellent agreement.

As follow-up experiments, the PLDOS can be measured using a different polarization direction by changing the local source and detector in the experimental setup. Changing the source and detector would allow for the experimental determination of the LDOS by measuring the PLDOS for the three orthogonal polarizations.

Furthermore, the setup can be operated on a slightly different configuration to be able to move the emitter and detector independently. This addition can extend the understanding of complex photonic structures by combining the determination of the PLDOS with the cross-density of optical states (CDOS). The CDOS is a measure of the intrinsic spatial coherence of the structures and is an important quantity in the design and optimization of photonic systems.^41^ PLDOS and CDOS complement each other since the (P)LDOS measures the number of optical states at a given location, defining the decay rate of a source at this location, while the CDOS measures the number of states connecting two distant points, defining the extended spatial coherence of point sources by coupling with the surrounding medium.

## Supporting Information Available

Experimental setup, fabrication of gold mirror and gold rod, gold rod total-field/scattered-field simulation, and additional gold rod measurements (PDF)

## References

[ref1] Purcell E. M. Spontaneous emission probabilities at radio frequencies. Phys. Rev. 1946, 69, 681.

[ref2] Yablonovitch E. Inhibited Spontaneous Emission in Solid-State Physics and Electronics. Phys. Rev. Lett. 1987, 58, 2059–2062. 10.1103/physrevlett.58.2059. 10034639

[ref3] Fang H. H.; Han B.; Robert C.; Semina M. A.; Lagarde D.; Courtade E.; Taniguchi T.; Watanabe K.; Amand T.; Urbaszek B.; Glazov M. M.; Marie X. Control of the Exciton Radiative Lifetime in van der Waals Heterostructures. Phys. Rev. Lett. 2019, 123, 067401 10.1103/physrevlett.123.067401. 31491178

[ref4] Barnes W. L.; Horsley S. A.; Vos W. L. Classical antennas, quantum emitters, and densities of optical states. J. Opt. 2020, 22, 073501 10.1088/2040-8986/ab7b01.

[ref5] Barnes W. L. Fluorescence near interfaces: The role of photonic mode density. J. Mod. Opt. 1998, 45, 661–699. 10.1080/09500349808230614.

[ref6] Chance R.; Prock A.; Silbey R. Molecular fluorescence and energy transfer near interfaces. Adv. Chem. Phys. 1978, 37, 1–65. 10.1002/9780470142561.ch1.

[ref7] Amos R. M.; Barnes W. L. Modification of the spontaneous emission rate of Eu^3+^ ions close to a thin metal mirror. Phys. Rev. B 1997, 55, 7249–7254. 10.1103/physrevb.55.7249.

[ref8] Ruppin R.; Martin O. J. Lifetime of an emitting dipole near various types of interfaces including magnetic and negative refractive materials. J. Chem. Phys. 2004, 121, 11358–11361. 10.1063/1.1812742. 15634093

[ref9] Lunnemann P.; Koenderink A. F. The local density of optical states of a metasurface. Sci. Rep. 2016, 6, 20655–20657. 10.1038/srep20655. 26868601 PMC4751612

[ref10] Tsakmakidis K. L.; Boyd R. W.; Yablonovitch E.; Zhang X. Large spontaneous-emission enhancements in metallic nanostructures: towards LEDs faster than lasers [Invited]. Opt. Express 2016, 24, 17916–17927. 10.1364/oe.24.017916. 27505759

[ref11] Andrew P.; Barnes W. L. Förster Energy Transfer in an Optical Microcavity. Science 2000, 290, 785–788. 10.1126/science.290.5492.785. 11052938

[ref12] Deeb C.; Guo Z.; Yang A.; Huang L.; Odom T. W. Correlating Nanoscopic Energy Transfer and Far-Field Emission to Unravel Lasing Dynamics in Plasmonic Nanocavity Arrays. Nano Lett. 2018, 18, 1454–1459. PMID: 29369639 10.1021/acs.nanolett.7b05223. 29369639

[ref13] Silveiro I.; Manjavacas A.; Thongrattanasiri S.; García de Abajo F. J. Plasmonic energy transfer in periodically doped graphene. New J. Phys. 2013, 15, 033042 10.1088/1367-2630/15/3/033042.

[ref14] Blum C.; Zijlstra N.; Lagendijk A.; Wubs M.; Mosk A. P.; Subramaniam V.; Vos W. L. Nanophotonic Control of the Förster Resonance Energy Transfer Efficiency. Phys. Rev. Lett. 2012, 109, 203601 10.1103/physrevlett.109.203601. 23215487

[ref15] Wubs M.; Vos W. L. Förster resonance energy transfer rate in any dielectric nanophotonic medium with weak dispersion. New J. Phys. 2016, 18, 053037 10.1088/1367-2630/18/5/053037.

[ref16] Alber G.; Bernád J. Z.; Stobińska M.; Sánchez-Soto L. L.; Leuchs G. QED with a parabolic mirror. Phys. Rev. A 2013, 88, 023825 10.1103/physreva.88.023825.

[ref17] Lozano G.; Louwers D. J.; Rodríguez S. R.; Murai S.; Jansen O. T.; Verschuuren M. A.; Gómez Rivas J. Plasmonics for solid-state lighting: Enhanced excitation and directional emission of highly efficient light sources. Light: Sci. Appl. 2013, 2, e66 10.1038/lsa.2013.22.

[ref18] Barnes W. L.; Björk G.; Gérard J. M.; Jonsson P.; Wasey J. A.; Worthing P. T.; Zwiller V. Solid-state single photon sources: Light collection strategies. Eur. Phys. J. D 2002, 18, 197–210. 10.1140/epjd/e20020024.

[ref19] Hoi I. C.; Kockum A. F.; Tornberg L.; Pourkabirian A.; Johansson G.; Delsing P.; Wilson C. M. Probing the quantum vacuum with an artificial atom in front of a mirror. Nat. Phys. 2015, 11, 1045–1049. 10.1038/nphys3484.

[ref20] Othman M. A.; Yazdi F.; Figotin A.; Capolino F. Giant gain enhancement in photonic crystals with a degenerate band edge. Phys. Rev. B 2016, 93, 024301 10.1103/physrevb.93.024301.

[ref21] Galisteo-López J. F.; Lozano G. Nanophotonics for current and future white light-emitting devices. J. Appl. Phys. 2021, 130, 200901 10.1063/5.0065825.

[ref22] Saive R. Light trapping in thin silicon solar cells: A review on fundamentals and technologies. Prog. Photovoltaics Res. Appl. 2021, 29, 1125–1137. 10.1002/pip.3440.

[ref23] Langguth L.; Fleury R.; Alù A.; Koenderink A. F. Drexhage’s Experiment for Sound. Phys. Rev. Lett. 2016, 116, 224301 10.1103/physrevlett.116.224301. 27314719

[ref24] Ohtsuki T.; Yuki H.; Muto M.; Kasagi J.; Ohno K. Enhanced Electron-Capture Decay Rate of ^7^Be Encapsulated in C_60_ Cages. Phys. Rev. Lett. 2004, 93, 112501 10.1103/physrevlett.93.112501. 15447332

[ref25] Drexhage K. H. Influence of a dielectric interface on fluorescence decay time. J. Lumin. 1970, 1–2, 693–701. 10.1016/0022-2313(70)90082-7.

[ref26] Vaskin A.; Kolkowski R.; Koenderink A. F.; Staude I. Light-emitting metasurfaces. Nanophotonics 2019, 8, 1151–1198. 10.1515/nanoph-2019-0110.

[ref27] Walther H.; Varcoe B. T.; Englert B.-G.; Becker T. Cavity quantum electrodynamics. Rep. Prog. Phys. 2006, 69, 1325–1382. 10.1088/0034-4885/69/5/r02.

[ref28] Novotny L.; Hecht B. Principles of Nano-Optics; 2nd ed.; Cambridge University Press, 2012.

[ref29] Sprik R.; Tiggelen B. A. v.; Lagendijk A. Optical emission in periodic dielectrics. Europhys. Lett. 1996, 35, 265–270. 10.1209/epl/i1996-00564-y.

[ref30] Koenderink A. F.; Bechger L.; Schriemer H.; Lagendijk A.; Vos W. L. Broadband fivefold reduction of vacuum fluctuations probed by dyes in photonic crystals. Phys. Rev. Lett. 2002, 88, 143903 10.1103/physrevlett.88.143903. 11955150

[ref31] Lodahl P.; Floris van Driel A.; Nikolaev I. S.; Irman A.; Overgaag K.; Vanmaekelbergh D.; Vos W. L. Controlling the dynamics of spontaneous emission from quantum dots by photonic crystals. Nature 2004, 430, 654–657. 10.1038/nature02772. 15295594

[ref32] Kühn S.; Håkanson U.; Rogobete L.; Sandoghdar V. Enhancement of single-molecule fluorescence using a gold nanoparticle as an optical nanoantenna. Phys. Rev. Lett. 2006, 97, 017402 10.1103/physrevlett.97.017402. 16907406

[ref33] Anger P.; Bharadwaj P.; Novotny L. Enhancement and quenching of single-molecule fluorescence. Phys. Rev. Lett. 2006, 96, 113002 10.1103/physrevlett.96.113002. 16605818

[ref34] Muskens O.; Giannini V.; Sánchez-Gil J. A.; Gómez Rivas J. Strong enhancement of the radiative decay rate of emitters by single plasmonic nanoantennas. Nano Lett. 2007, 7, 2871–2875. 10.1021/nl0715847. 17683156

[ref35] Ropp C.; Cummins Z.; Nah S.; Fourkas J. T.; Shapiro B.; Waks E. Nanoscale imaging and spontaneous emission control with a single nano-positioned quantum dot. Nat. Commun. 2013, 4, 1447 10.1038/ncomms2477. 23385591

[ref36] Wertz E.; Isaacoff B. P.; Flynn J. D.; Biteen J. S. Single-Molecule Super-Resolution Microscopy Reveals How Light Couples to a Plasmonic Nanoantenna on the Nanometer Scale. Nano Lett. 2015, 15, 2662–2670. PMID: 25799002 10.1021/acs.nanolett.5b00319. 25799002

[ref37] Schell A. W.; Engel P.; Werra J. F. M.; Wolff C.; Busch K.; Benson O. Scanning Single Quantum Emitter Fluorescence Lifetime Imaging: Quantitative Analysis of the Local Density of Photonic States. Nano Lett. 2014, 14, 2623–2627. PMID: 24694035 10.1021/nl500460c. 24694035

[ref38] García de Abajo F. J.; Kociak M. Probing the photonic local density of states with electron energy loss spectroscopy. Phys. Rev. Lett. 2008, 100, 106804 10.1103/physrevlett.100.106804. 18352220

[ref39] van Hoof N. J.; Abujetas D. R.; ter Huurne S. E.; Verdelli F.; Timmermans G. C.; Sánchez-Gil J. A.; Rivas J. G. Unveiling the Symmetry Protection of Bound States in the Continuum with Terahertz Near-Field Imaging. ACS Photonics 2021, 8, 3010–3016. 10.1021/acsphotonics.1c00937. 34692900 PMC8532159

[ref40] ter Huurne S.; Abujetas D. R.; van Hoof N.; Sanchez-Gil J. A.; Gómez Rivas J. Direct Observation of Lateral Field Confinement in Symmetry-Protected THz Bound States in the Continuum. Adv. Opt. Mater. 2023, 11, 2202403 10.1002/adom.202202403.

[ref41] Cazé A.; Pierrat R.; Carminati R. Spatial coherence in complex photonic and plasmonic systems. Phys. Rev. Lett. 2013, 110, 063903 10.1103/physrevlett.110.063903. 23432244

