# Supplementary material for: Direct Measurement of the Local Density of Optical States in the Time Domain
Source: ACS Photonics. 2023 Aug 1;10(8):2980–6. doi: 10.1021/acsphotonics.3c00781 (PMC10436706; doi:10.1021/acsphotonics.3c00781)
Supplement: Supplementary file 1 — ph3c00781_si_001.pdf [file ph3c00781_si_001.pdf]

Supporting information

Direct Measurement of the Local Density of Optical States in the Time-Domain

Stan E.T. ter Huurne,[†] Djero B.L. Peeters,[†] Jose A. Sánchez-Gil,[‡] and Jaime Gómez Rivas[*,†]

[†]*Department of Applied Physics and Science Education, and Eindhoven Hendrik Casimir Institute, Eindhoven University of Technology, P.O. Box 513, 5600 MB Eindhoven, The Netherlands*

[‡]*Instituto de Estructura de la Materia, Consejo Superior de Investigaciones Científicas (IEM-CSIC), Serrano 121, Madrid 28006, Spain*

E-mail: j.gomez.rivas@tue.nl

SI.1 Experimental Setup Schematic

A schematic of the double near-field THz probe microscope is presented in Fig. S1. A femtosecond laser pulse is split with a beam splitter. One optical path is directed at the tip of the detector probe with mirrors. The other optical path is varied in length by a delay stage, after which the laser is fiber coupled and then focused onto the emitter probe. To control the distance between the probe and the sample, we use a MTS-65 linear stage from Physik Instrument. With this stage the position of the sample can be moved with an accuracy of 100 nm with respect to the MPCAs. To determine the distance, we make use of a camera. We can see both the sample and the antennas simultaneously and we move them closer together until we can visibly see that the antennas start touching the sample. From that position, we can increase the distance with 100 nm accuracy.

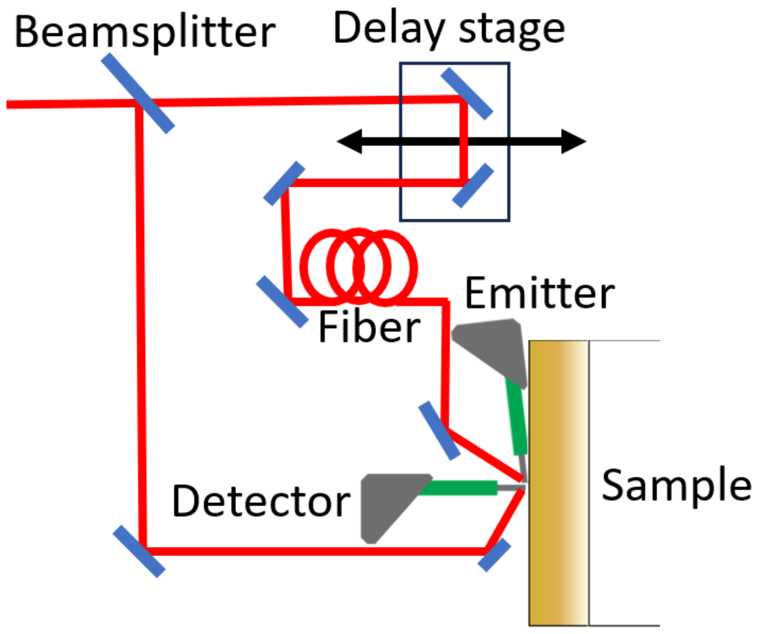

Figure S1: Schematic of the double near-field THz probe microscope.

SI.2 Fabrication of Gold Mirror and Gold Rod

For the fabrication of the gold mirror and the gold rod resonator, the quartz substrate was cleaned in an acetone bath for 5 minutes, followed by a bath of isopropyl alcohol for 5 minutes, after which it was rinsed with dH2O. The substrate was activated with an Ion Wave Stripper using oxygen plasma at 600 W for 5 minutes. To improve adhesion of the photoresist, the substrate was vapor-coated with HDMS. A negative photoresist, MaN-440, was spin-coated at 3000 rpm for 30 seconds with an acceleration of 1000 rpm/s. The photoresist film was soft baked at 100 °C for 2 minutes, after which the final thickness was 2 μm. Using a Karl Suss MA-6 optical lithography machine, the substrate was exposed for 100 seconds at 365 nm. This process was repeated 3 times with 10 seconds interval between the exposures. To ensure a resolution of more than 1.5 μm, the contact mode was set to 'hard'. The exposed sample was developed for 90 seconds in a MaD-532s developer after which it was rinsed for 2 minutes in dH2O. Using a BVR2008FC Electron Beam Evaporator, 2 nm titanium and 100 nm gold were deposited with evaporation rates of 0.5 nm/s and 1 nm/s, respectively. The lift-off was performed using acetone. After lift-off, the final step was rinsing the sample in dH2O for 2 minutes.

SI.3 Gold Rod Total-Field Scattered-Field Simulation

We have obtained the resonant response of a single gold rod of 200 μm by 40 μm in size, and with a height of 100 nm on a quartz substrate using FDTD simulations. The permittivities used for the gold and quartz are ϵ_{gold}=-1.54$\cdot 10^5$+8$\cdot 10^5$i and ϵ_{quartz}=4.[1,2] A total-field scattered-field (TFST) source was used to determine the scattering efficiency. This source is typically used to investigate the scattering of single particles illuminated by a plane wave. The TFSF source separates the computation volume into two regions: the total field region, which includes the incident wave plus the scattered field, and the scattered field region, which includes only the scattered field. The simulated scattering efficiency using the TFSF

source for radiation polarized along the long axis of the gold rod is shown in Fig. S2. The scattering efficiency is defined as the scattering cross-section normalized to the geometrical cross-section. The rod shows a broad resonant response centered at 0.4 THz which corresponds to the $\lambda/2$ resonance.

The PLDOS of the rod was determined by simulating the rate of energy dissipation of a point dipole next to the rod and normalizing it by the power that the dipole would radiate in a homogeneous medium. The dipole was moved over the surface to retrieve the PLDOS maps shown in the manuscript.

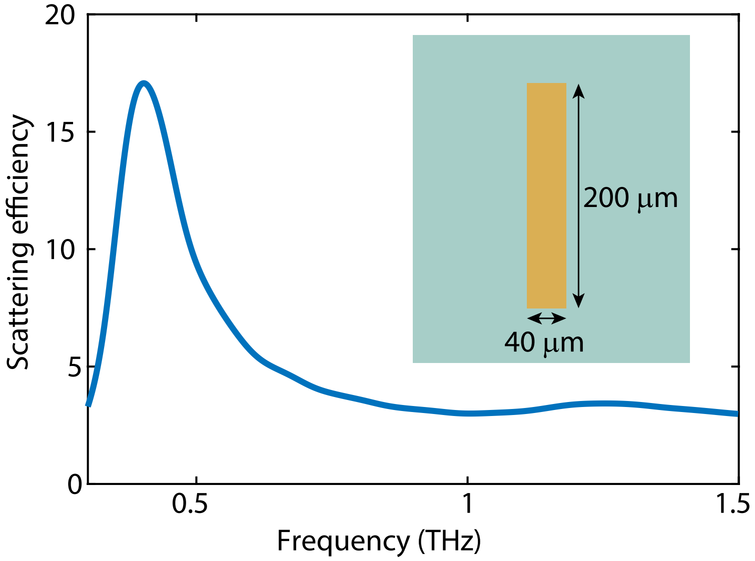

Figure S2: Scattering efficiency of a single gold rod of 200 μm by 40 μm with a height of 100 nm on a quartz substrate (schematically shown in the inset), obtained by total-field scattered-field FDTD simulations.

SI.4 Additional Gold Rod Measurements

A map of a linescan of transients along the long axis of the gold rod is shown in Fig. S3a. The non-symmetric response with respect to the rod is attributed to a slight misalignment between the source and detector along the Y-direction. This offset is required to minimize the influence of reflected and scattered laser light used for excitation and detection of the THz pulses. A map of the frequency dependent normalized PLDOS along the long axis of

the rod is shown in Fig. S3b. The two edges show an enhancement with a shifted frequency: the bottom edge shows a at 0.4 THz, while the top edge shows a peak at 0.3 THz.

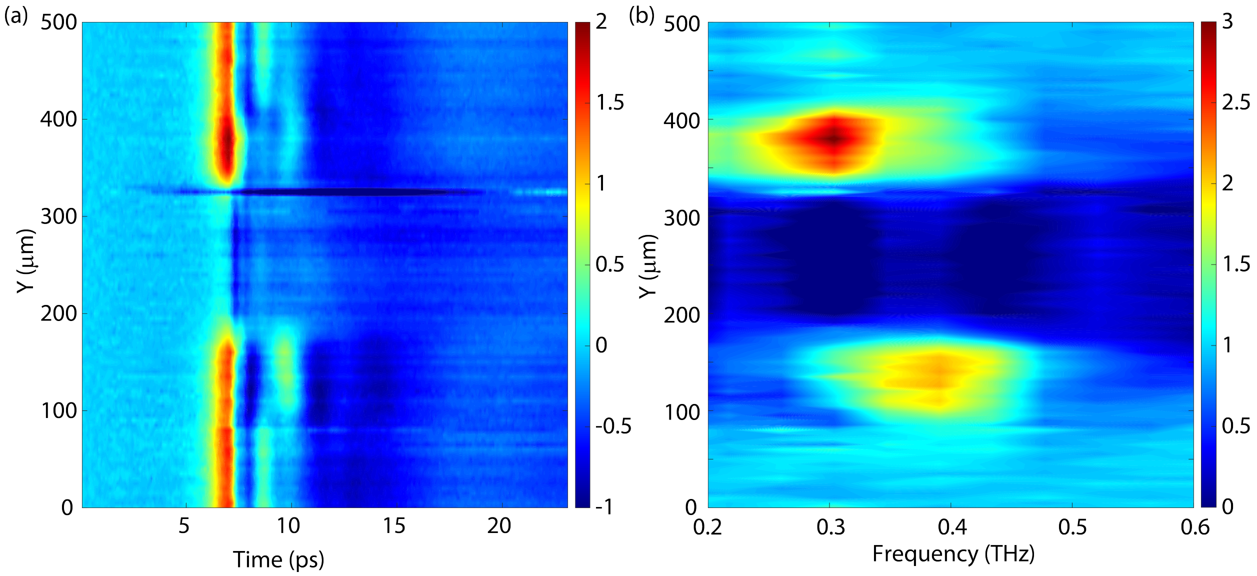

Figure S3: (a) Electric field amplitude as a function of time along the long axis of the rod. (b) PLDOS normalized by the PLDOS of the quartz substrate as a function of frequency along the direction of the long axis of the rod.

References

(1) Naftaly, M.; Miles, R. E. Terahertz time-domain spectroscopy of silicate glasses and the relationship to material properties. Journal of Applied Physics Journal of Applied Physics Applied Physics Letters Journal of Applied Physics **2007**, 102, 43505–31901.

(2) Giannini, V.; Fernández-Domínguez, A. I.; Sonnefraud, Y.; Roschuk, T.; Fernández-García, R.; Maier, S. A. Controlling light localization and light-matter interactions with nanoplasmonics. Small **2010**, 6, 2498–2507.
